# Improvement of the reverse tetracycline transactivator by single amino acid substitutions that reduce leaky target gene expression to undetectable levels

**DOI:** 10.1038/srep27697

**Published:** 2016-06-21

**Authors:** Ian J. Roney, Adam D. Rudner, Jean-François Couture, Mads Kærn

**Affiliations:** 1 Ottawa Institute of Systems Biology, University of Ottawa, Ottawa, K1H8L1, Canada; 2 Department of Cellular and Molecular Medicine, University of Ottawa, Ottawa, K1H8L1, Canada; 3 Department of Biochemistry, Microbiology and Immunology, University of Ottawa, Ottawa, K1H8L1, Canada; 4 Department of Physics, University of Ottawa, Ottawa, K1N6N5, Canada; 5 Cancer Therapeutics Program, Ottawa Hospital Research Institute, Ottawa, K1H8L6, Canada

## Abstract

Conditional gene expression systems that enable inducible and reversible transcriptional control are essential research tools and have broad applications in biomedicine and biotechnology. The reverse tetracycline transcriptional activator is a canonical system for engineered gene expression control that enables graded and gratuitous modulation of target gene transcription in eukaryotes from yeast to human cell lines and transgenic animals. However, the system has a tendency to activate transcription even in the absence of tetracycline and this leaky target gene expression impedes its use. Here, we identify single amino-acid substitutions that greatly enhance the dynamic range of the system in yeast by reducing leaky transcription to undetectable levels while retaining high expression capacity in the presence of inducer. While the mutations increase the inducer concentration required for full induction, additional sensitivity-enhancing mutations can compensate for this effect and confer a high degree of robustness to the system. The novel transactivator variants will be useful in applications where tight and tunable regulation of gene expression is paramount.

The reverse tetracycline transactivator (rtTA) combines a variant of the strong DNA binding domain from the TetR regulator of the bacterial tetracycline resistance transposon with a highly efficient viral transcriptional activation domain^1^. Upon the addition of tetracycline, rtTA undergoes a conformational change that increases its affinity for a unique 19 base-pair DNA binding site and enhances the recruitment of the machinery required for transcription^1^. The rtTA system offers many advantages because it enables graded and reversible transcriptional control using a non-toxic inducer, the antibiotic doxycycline, that has few pleiotropic effects^2^. A disadvantage of the system is that the rtTA protein retains some affinity for its DNA binding site in the absence of inducer resulting in leaky transcription of target genes^3^,^4^,^5^,^6^,^7^,^8^,^9^.

Two properties of rtTA have been targets for extensive optimization: increasing the sensitivity to doxycycline and increasing its dynamic range by reducing its basal activity in the un-induced state. Directed evolution has proven to be a powerful approach to improve sensitivity^10^,^11^ but this does not select for variants with decreased basal activity. Strategies that have been used to reduce the leaky transcription include combining rtTA with a tetracycline-responsive repressor^3^,^12^,^13^, optimization of the rtTA target promoter^5^,^6^, and the introduction of positive feedback in the control of rtTA gene transcription^8^. Yet, the problem of high leaky transcription persists and continues to complicate the uses of the rtTA system. For example, when used in mammalian cells, it has been reported that multiple clones need to be examined to find one that has low target gene expression in the absence of inducer^6^. In a recent implementation of an rtTA system controlling CRISPR/Cas9, up to 33% of clones may have off-doxycycline mutagenesis caused by leaky Cas9 expression^14^.

## Results

**Figure 1:**
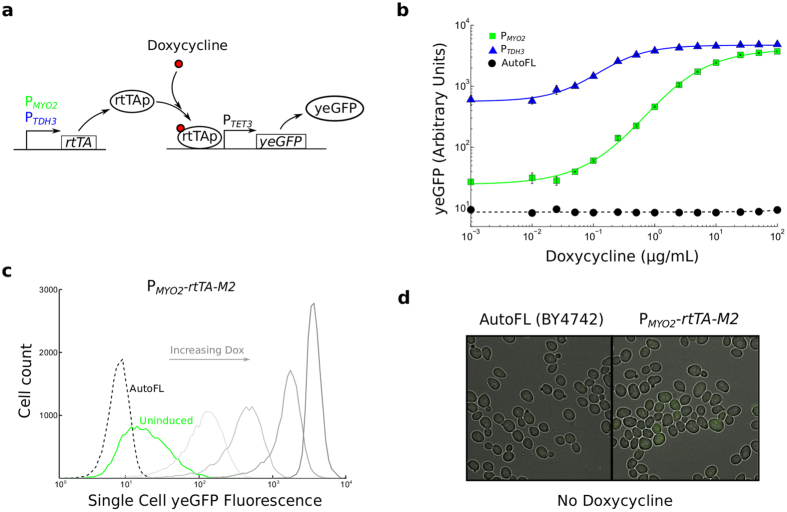
rtTA-M2 has significant activity in the absence of doxycycline induction. (**a**) Schematic of the experimental setup where rtTA-M2 is expressed from the *P_MYO2_* or *P_TDH3_* constitutive promoters and *yeGFP* is expressed from a tet-responsive promoter. (**b**) Doxycycline dose-dependent activation of rtTA-M2 expressed from either *P_MYO2_* or *P_TDH3_* (mean +/− s.e.m of three technical replicates). AutoFL is wildtype BY4742 yeast cells. (**c**) Distributions of single-cell fluorescence obtained from cells carrying the *P_MYO2_-rtTA* system at different doxycycline concentrations. Progressively darker shades of grey correspond to 0.25, 1, 5, and 100 *μ*g/mL of doxycycline respectively. AutoFL is wildtype BY4742 yeast cells (**d**) Merged brightfield-fluorescence images of wildtype BY4742 yeast cells or uninduced cells carrying the *P_MYO2_-rtTA* system.

Tetracycline inducible systems have been used extensively in yeast to study the control of gene transcription in single cells^15^,^16^,^17^,^18^,^19^,^20^,^21^,^22^. To use rtTA in this context, we created two strong doxycycline-responsive promoters with either three (*P_TET3_*) or four (*P_TET4_*) rtTA binding sites, and used the optimized rtTA-M2 variant^7^ to control the expression of yeast-enhanced green fluorescent protein (*yeGFP*)^23^ from these promoters (Fig. 1a). The rtTA/*P_TET_-yeGFP* expression cassette was integrated in a single copy into the yeast genome. The M2 variant is identical to the variant found in ClonTech’s Tet-ON Advanced expression systems.

The problem of leaky target gene transcription is especially profound when rtTA is expressed at high levels. This is illustrated in Fig. 1b which shows *P_TET3_* doxycycline dose response curves when rtTA-M2 is expressed from the strong *P_TDH3_* promoter. Fluorescence was very high when cells were exposed to saturating amounts of doxycycline. Nonetheless, because of leaky transcription from the *P_TET3_* promoter, the dynamic range of the system was rather poor with the maximal expression ~17 times greater at full induction compared to the absence of doxycycline.

Consistent with a model where the transactivator has significant activation potential in its un-induced state, expression of rtTA-M2 from the weak *P_MYO2_* promoter substantially reduced fluorescence in the absence of doxycycline. Although full saturation was not observed with this system, the reduction of leaky expression resulted in a drastic improvement in dynamic range and induction with doxycycline resulted in an ~200 fold increase in fluorescence. However, despite this improvement, both flow cytometry data (Fig. 1c) and fluorescence microscopy data (Fig. 1d) indicated that un-induced rtTA-M2 causes significant reporter gene expression even when the transactivator is expressed from a weak promoter.

**Figure 2:**
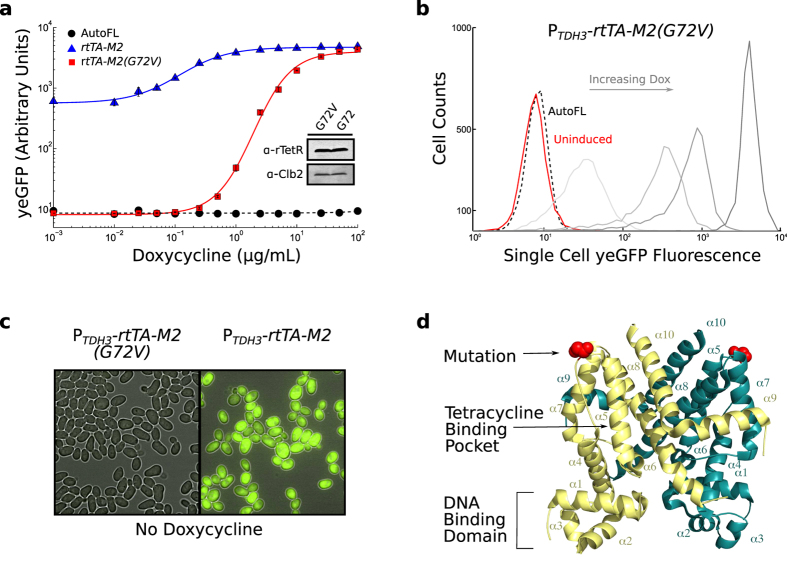
Mutation of glycine 72 in rtTA-M2 diminishes basal activity to undetectable levels. (**a**) Doxycycline dose-dependent activation of rtTA-M2 variants expressed from *P_TDH3_* (mean +/− s.e.m of three technical replicates). AutoFL is wildtype BY4742 yeast cells. In the inset, a western blot (cropped) comparing protein abundances of both rtTA-M2 and the G72V mutant. All samples were prepared and run under identical experimental conditions. (**b**) Distributions of single-cell fluorescence obtained from cells carrying the *P_TDH3_-rtTA*(*G72V*) system at different doxycycline concentrations. AutoFL is wildtype BY4742 yeast cells. Progressively darker shades of grey correspond to 1, 2.5, 5 and 100 *μ*g/mL of doxycycline respectively. (**c**) Merged brightfield-fluorescence images of uninduced cells carrying the *P_TDH3_-rtTA-M2* or *P_TDH3_-rtTA*(*G72V*) systems. (**d**) Cartoon representation of TetR dimer in which each protomer is colored different (yellow and turquoise). The secondary structure is labeled and G72 is shown as a sphere.

During the initial testing of twelve clones carrying the *P_TDH3_-rtTA-M2* system, we serendipidously discovered that one of them emitted unexpectedly low fluorescence in the absence of doxycycline but almost normal fluorescence at full induction (Fig. 2a). Subsequent sequencing identified a single nucleotide mutation, guanine to thymine, that changes a glycine (GGG) to a valine (GTG) at residue 72 within the transactivator protein. Western blot analysis indicated that this substitution does not affect protein abundance (Fig. 2a, Insert, see Supplementary Fig. S1 for details), and reconstructing the expression system confirmed that the single guanine to thymine mutation is responsible for the changes seen in the doxycycline dose response (data not shown).

The *P_TDH3_-rtTA-M2*(*G72V*) system has a remarkable dynamic range and showed an ~500 fold increase in fluorescence intensity when cells were induced with high doxycycline compared to no induction (Fig. 2b). Moreover, the fluorescence emission detected by flow cytometry is indistinguishable from cellular auto-fluorescence in the absence of doxycycline, and the variant preserves the graded induction response of the original transactivator. Moreover, reporter expression from the un-induced rtTA-M2(G72V) strain was undetectable by fluorescence microscopy even under instrument settings where the un-induced rtTA-M2 strain gave a strong saturating signal (Fig. 2c).

To gain insight into how the single mutation can have such a drastic impact on the dynamic range of rtTA-M2(G72V), we investigated where the residue was located in the context of the three dimensional structure of the protein. The structure of rtTA-M2 or other rtTA variants has not been solved. However, with 203 out of 207 amino acids in the TetR domain of rtTA being preserved^7^, the high resolution structure of TetR^24^ gives insight into the structure of the rtTA DNA binding domain. In the TetR structure, the glycine residue 72 maps to a flexible loop located between *α*-helices *α*4 and *α*5, a region bridging the repressor DNA binding domain (*α*1–*α*3) and its tetracycline binding region (*α*5–*α*9)^24^,^25^ (Fig. 2d). This suggests that the substitution, which introduces a non-polar side-chain, triggers long range conformational changes that ultimately affect the activity of the transactivator, presumably by rigidifying its tertiary structure.

**Figure 3:**
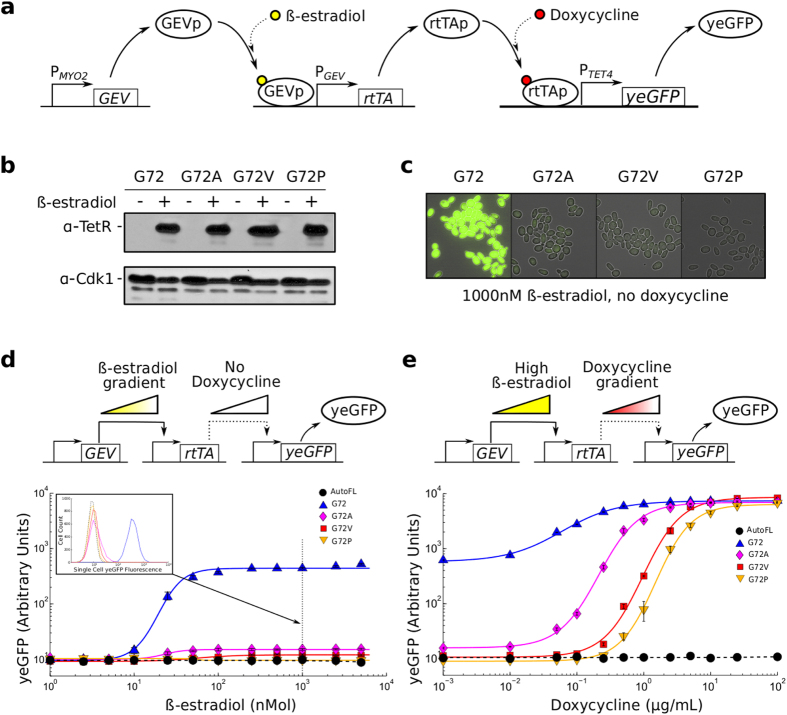
Non-polar side chains at residue 72 is a key determinant of basal activity. (**a**) Schematic of the gene network where rtTA-M2 variants are expressed from a *β*-estradiol inducible promoter and *yeGFP* is expressed from a tet-responsive promoter. (**b**) Western blot (cropped) comparing protein abundances of all four rtTA variants when induced by 1000 nM of *β*-estradiol or no induction. All samples were prepared and run under identical experimental conditions. (**c**) Merged brightfield-fluorescence images of all four rtTA variants induced by 1000 nM *β*-estradiol with no doxycycline induction. (**d**) The basal activity of rtTA variants as a function of their *β*-estradiol-dependent expression level (mean +/− s.e.m of three technical replicates). In the inset, histograms of reporter expression for the different rtTA variants. (**e**) Doxycycline dose-dependent activation of rtTA-M2 variants expressed ubiquitously in 1000 nM *β*-estradiol (mean +/− s.e.m of three technical replicates).

To examine this hypothesis, we created two additional variants in which the glycine residue was mutated into alanine or proline. These mutations preserve the non-polar nature of the valine residue but introduce side chains of varying size. We anticipated that the single methyl side-chain of alanine would be less effective in preventing uninduced activity than the large cyclical side chain of proline. We also created a *β*-estradiol inducible expression system^26^ to gain direct control of rtTA-M2 expression, and used the promoter with an extra TetR binding site, *P_TET4_*, to enhance the ability of rtTA to bind DNA. The expression system is illustrated schematically in (Fig. 3a). We confirmed by western blot analysis that the four rtTA variants are expressed at similar levels at full *β*-estradiol induction (Fig. 3b, see Supplementary Fig. S2 for details), and that the fluorescence of a strain lacking rtTA has reporter expression that is indistinguishable from a wildtype BY4742 yeast strain, demonstrating that there is no background reporter expression from *P_TET4_* (Supplementary Fig. S3).

As expected, the alanine variant showed higher leaky expression than the valine variant, and the proline variant had fluorescence that was indistinguishable from cellular auto-fluorescence as measured by flow cytometry. This is apparent from fluorescence microscopy images obtained with instrument settings where the strain harbouring the original M2 variant gives a strong fluorescence signal at full *β*-estradiol induction (Fig. 3c), and from fluorescence measurements by flow cytometry at varying *β*-estradiol induction (Fig. 3d).

Remarkably, the amino acid substitutions that significantly reduce basal reporter gene expression have minimal effect on rtTA transcriptional activation at full *β*-estradiol and doxycycline induction. This is illustrated in Fig. 3E, which displays the dose-response curves for the four G72-M2 variants measured when doxycycline is varied at full *β*-estradiol induction. However, the amino acid substitutions impact doxycycline sensitivity. In our experiments (Fig. 3e), the M2 variant had the highest sensitivity with a half maximal effective concentration (EC50) of ~0.06 *μ*g/mL while the alanine variant (EC50 of ~0.2 *μ*g/mL), the valine variant (EC50 of ~1.0 *μ*g/mL) and the proline variant (EC50 of ~1.5 *μ*g/mL) required progressively higher doxycycline concentrations to reach maximal expression capacity.

To counter the effect of the G72 mutation on doxycycline sensitivity, we introduced additional mutations in the TetR domain that were recently shown to increase the sensitivity to doxycycline^10^,^11^,^27^. We specifically examined the effect of introducing the sensitivity enhancing (SE) mutations V9I, F67S, F86Y and R171K.

**Figure 4:**
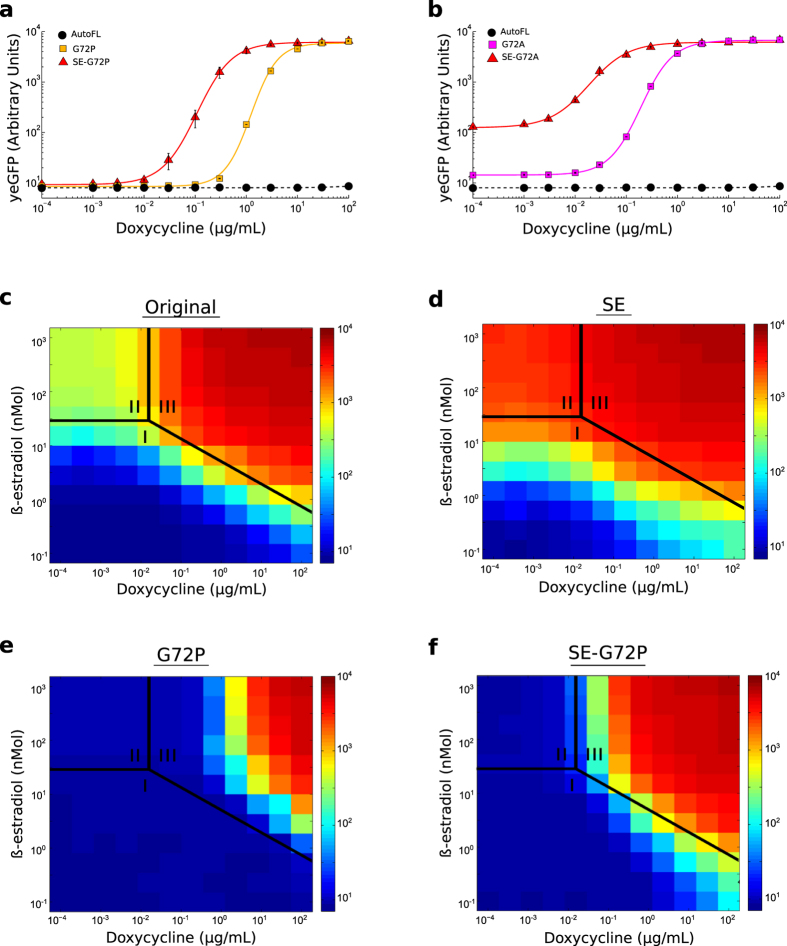
Doxycycline sensitivity of the novel rtTA variants can be improved by the addition of sensitivity enhancing mutations. (**a**,**b**) Doxycycline dose-dependent activation of rtTA variants expressed ubiquitously in 1000 nM *β*-estradiol (mean +/− s.e.m of three technical replicates). (**c–f**) Heatmaps displaying reporter expression (Arbitrary units) as a function of *β*-estradiol-dependent transactivator expression and doxycycline induction.

Figure 4A,B demonstrate that the SE mutations improve the doxycycline sensitivity of the SE-G72P and SE-G72A rtTA M2 variants. When the rtTA variants are expressed at high levels from the fully activated *β*-estradiol inducible promoter (Fig. 3a), the introduction of the SE mutations reduces the doxycycline EC50 from ~1.5 *μ*g/mL to ~0.1 *μ*g/mL for the G72P variant (Fig. 4a) and from ~0.2 *μ*g/mL to ~0.02 *μ*g/mL for the G72A variant (Fig. 4b). This effect occurred without an appreciable change in reporter expression under full doxycycline induction, and did not compromise the dynamic range of the SE-G72P variant. Interestingly, however, the SE mutations caused a significant loss of dynamic range for the SE-G72A variant by increasing reporter gene expression in the absence of doxycycline.

To further explore the relationship between the level of rtTA expression, doxycycline EC50 and basal activity, we examined the effect on reporter gene expression of simultaneously varying *β*-estradiol and doxycycline induction. We investigated four different M2 variants; the original variant, the orginal variant with the SE mutations, the G72P variant and the SE-G72P variant.

The two-dimensional dose-response surface for the original M2 variant contains three distinct regions of reporter expression corresponding to low, intermediate and high reporter gene expression. These regions are labeled I, II and III in Fig. 4c–f. Region III is where reporter expression is maximal, which depends on both rtTA expression level and the level of doxycycline induction. Region II is where reporter expression is relatively high even when doxycycline induction is low. Region I is where reporter expression is low due to low *β*-estradiol or low doxycycline induction.

The SE mutations alone (Fig. 4d) dramatically increase reporter expression in part of region I and the entire region II. This confirms that these mutations may enhance sensitivity in a narrow range of rtTA expression levels, but also cause a significant increase in the activity of rtTA in the un-induced state. Contrasting this, the G72P mutation alone (Fig. 4e) dramatically reduces reporter expression in the entire region II and in part of region III. This confirms that the profound effect of this mutation on rtTA activity in the un-induced state is associated with a general loss of doxycycline sensitivity.

Figure 4f shows the effect of combining the SE and the G72P mutations. Compared to the SE variant (Fig. 4d), adding the G72P mutation counters the increase in reporter expression caused by the SE mutations in region I and reduces reporter expression to undetectable levels in this region. Moreover, compared to the G72P variant (Fig. 4e), adding the SE mutations almost completely restores the loss of reporter expression in region III. In other words, sensitivity is improved without introducing leaky target gene expression at any transactivator expression level.

## Discussion

We have identified single amino acid substitutions in the widely used doxycycline-inducible transactivator (rtTA) that significantly improves dynamic range without compromising maximal expression capacity. Replacing a single glycine (G72) in the rtTA-M2 variant with residues that introduce non-polar side chains reduces its ability to activate transcription in the absence of doxycycline in a manner that depends on the size of the side chain. This suggests that the reduction in leaky target gene expression may be due to an increased rigidity of the tertiary structure of rtTA that reduces its DNA binding affinity in the absence of induction.

The mutations that reduce leaky target gene expression also reduce the sensitivity to doxycycline. This loss of sensitivity depends on the size of the side chain introduced at G72, suggesting a complex interplay between the rtTA DNA binding domain and its doxycycline binding pocket.

The loss of sensitivity could be a problem in applications where it is not possible, or not practicable, to use high doxycycline concentrations. We have demonstrated, however, that introducing additional mutations into the G72P M2 variant can improve the sensitivity to doxycycline without introducing leaky target gene expression. The variant with high sensitivity and undetectable leaky target gene expression carries five mutations (V9I, F67S, G72P, F86Y, R171K) in the TetR domain of rtTA-M2 and the G72P mutation appears to be critical. A G72A variant has higher doxycycline sensitivity (lower EC50) than the G72P variant, but also substantially higher activity in the absence of doxycycline. These observations suggest that the combined effect of mutations that impact rtTA DNA binding and doxycycline induction are highly nonlinear.

The rtTA expression system serves a critical role in biological and biomedical research as a means to precisely control target gene expression in a graded and gratuitous manner. It also has many potential applications in gene therapy. In most applications, an optimal inducible gene expression system has undetectable target gene expression in the absence of inducer and sufficiently high target gene expression at non-toxic and achievable inducer concentrations. Usually, there is a trade-off between low leakiness and high expression capacity^27^. Our sensitivity-enhanced G72P M2 variant retains low leakiness and high expression capacity over a broad range of expression levels in yeast. Such robustness of function may be particularly advantageous in experiments where transactivator expression levels may vary, such as transient transfection or random integration of an expression cassette. Although past studies have shown that other rtTA variants first characterized in yeast preserve their function when expressed in mammalian cell lines and transgenic animals, it will be important to further investigate if the improved function of the sensitivity-enhanced G72P M2 variant extends from yeast to higher organisms.

## Methods

### Strain construction

Overlap extension PCR was used to create all synthetic DNA constructs^28^. DNA constructs containing the yeGFP, GEV and rtTA parts were sequentially integrated into the genome at the *ADE2*, *GAL4* and *ADE4* loci respectively, of *S. cerevisiae* strain BY4742 (MAT*α*, his3Δ1, leu2Δ0, lysΔ0, ura3Δ0) (EUROSCARf). The *GAL1* promoter with the *GAL4* UAS sequence removed served as the starting material for constructing the tet-responsive promoters. The *P_TET3_* promoter was built by inserting tet operators at nucleotides −184, −267 and −351 from the *yeGFP* ATG. *P_TET4_* had an additional site at nucleotide −417 from the *yeGFP* ATG. A standard lithium acetate transformation protocol^29^ was used for integrating all constructs into the genome. Cells were selected after transformation on either YP media supplemented with 2% glucose, 2% adenine and appropriate antibiotic selection or on synthetic drop out media supplemented with 2% glucose and 2% adenine.

### Cell culture and media

Single yeast colonies from antibiotic or auxotrophic selection plates were inoculated in synthetic media supplemented with 2% glucose and 2% adenine. Cells were maintained in logarithmic growth phase prior to and during induction with doxycycline and/or *β*-estradiol. Cells were grown under inducing conditions for 6 hours before measuring yeGFP fluorescence by flow cytometry or microscopy.

### Flow cytometry acquisition and analysis

Yeast cultures were diluted 1 in 10 in 50 mM sodium citrate buffer prior to being sampled by the flow cytometer. An IntelliCyt HyperCyt autosampler attached to a Beckman Coulter CyAn ADP9 analyzer was used for the collection of all flow cytometry data. Yeast cells were gated with a small forward and side scatter gate (~40% of the population) to reduce variability from extrinsic sources. A 488 nM laser and a 530/40 band-pass filter were used to excite and detect yeGFP fluorescence. FCS files were analyzed by custom scripts created in MATLAB R2013a (Mathworks Inc, Natick, Massachusetts). Mean fluorescent intensity values and standard error values were calculated from this data.

### Fluorescent microscopy acquisition and analysis

Yeast cell cultures were concentrated by centrifugation at 4000RPM in an Eppendorf 5415D desktop centrifuge, re-suspended in sterile water, pipetted onto microscopy slides and immediately imaged. Fluorescent microscopy was performed with a Nikon Ti-E inverted fluorescent microscope with a Chroma HQ480/40x excitation and HQ535/50 m emission filter cube. NIS Elements software was used to merge brightfield and fluorescent images.

### Western blotting

Western blotting was completed as previously described^30^. The *α*TetR (Clontech), *α*CDK1 (Rabbit Polyclonal) and *α*CLB2 (Covance) antibodies were used at dilutions of 1/1000, 1/1000 and 1/4000 respectively.

### Protein structure modeling

The figure was generated using the structure of TETR(b) in complex with Minocycline and magnesium (4AC0.pdb)^24^ and the PyMOL^31^ software.

## Additional Information

**How to cite this article**: Roney, I. J. *et al*. Improvement of the reverse tetracycline transactivator by single amino acid substitutions that reduce leaky target gene expression to undetectable levels. *Sci. Rep.* **6**, 27697; doi: 10.1038/srep27697 (2016).

## Supplementary Material

Supplementary Information
