# Supplementary material for: Improvement of the reverse tetracycline transactivator by single amino acid substitutions that reduce leaky target gene expression to undetectable levels
Source: Sci Rep. 2016 Jun 21;6:27697. doi: 10.1038/srep27697 (PMC4914848; doi:10.1038/srep27697)
Supplement: Supplementary Information [file srep27697-s1.pdf]

Improvement of the reverse tetracycline transactivator by single amino acid substitutions that reduce leaky target gene expression to undetectable levels

Ian J. Roney[1,2], Adam D. Rudner[1,3], Jean-Francois Couture[1,3], and Mads Kærn[1,2,4,5*]

[1]Ottawa Institute of Systems Biology, University of Ottawa, Ottawa, K1H8L1, Canada.

[2]Department of Cellular and Molecular Medicine, University of Ottawa, Ottawa, K1H8L1, Canada.

[3]Department of Biochemistry, Microbiology and Immunology, University of Ottawa, Ottawa, K1H8L1, Canada.

[4]Department of Physics, University of Ottawa, Ottawa, K1N6N5, Canada

[5]Cancer Therapeutics Program, Ottawa Hospital Research Institute, Ottawa, K1H8L6, Canada

[*]correspondence should be addressed to M.K by email at: mkaern@uottawa.ca

The following blots were cropped to produce the inset of Figure 2a.

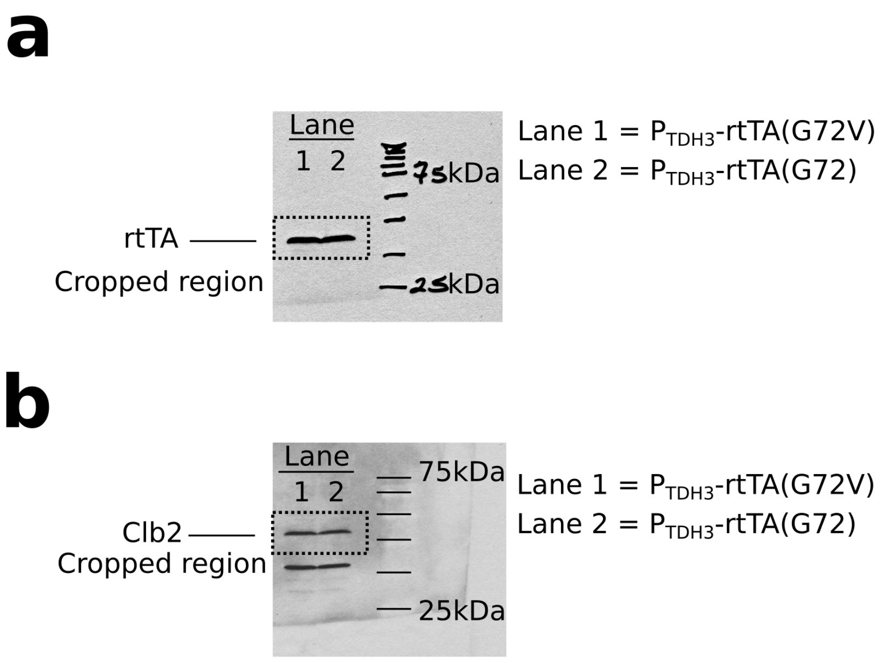

Supplemental figure S1. Glycine to valine substitution at residue 72 does not affect steady state protein levels. **a,** Western blot comparing the rtTA protein abundances of the rtTA-M2 and rtTA-M2(G72V) variants when expressed from theTDH3 promoter. **b,** Loading control comparing protein abundances of Clb2 in strains expressing the rtTA-M2 variant or rtTA-M2(G72V) variant.

The following blots were cropped to produce Figure 3B.

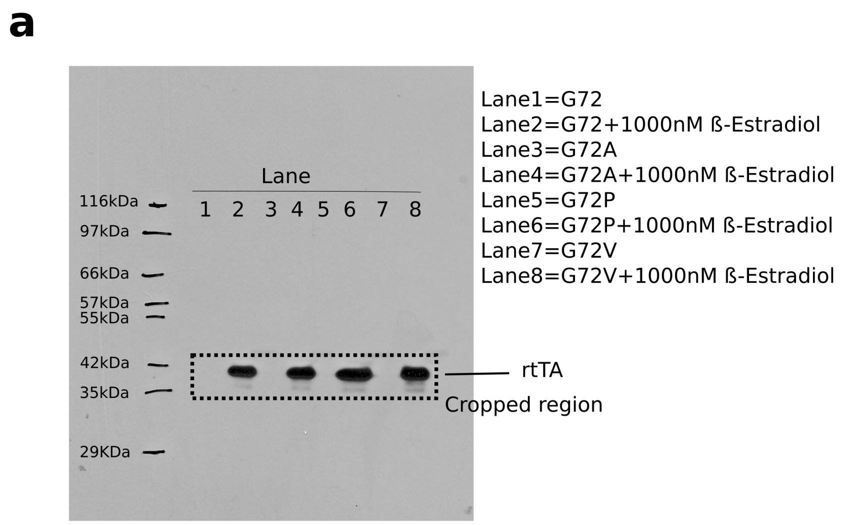

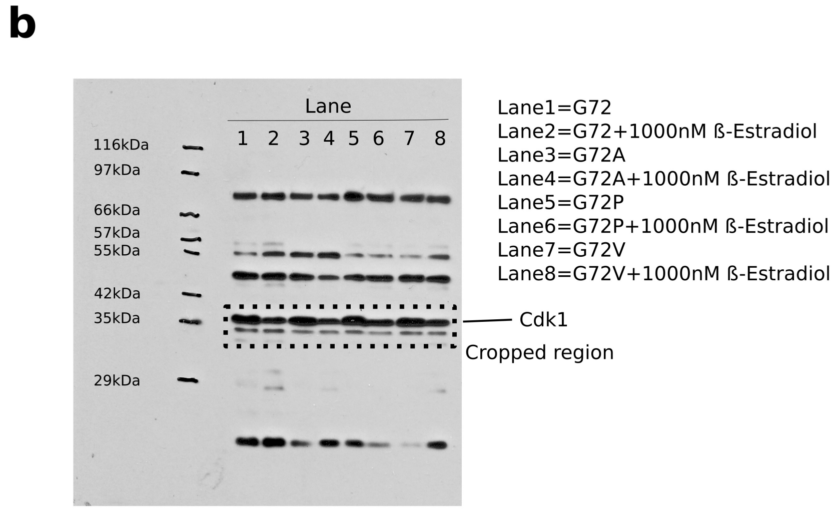

Supplemental Figure S2. Substitution of glycine for non-polar side chains at residue 72 does not affect steady state protein levels. **a,** Western blot comparing the rtTA protein abundances of all four rtTA variants when cells were induced by 1000nM of β-estradiol or no induction. **b,** Loading control comparing protein abundances of Cdk1 when cells expressing the four rtTA variants were induced by 1000nM of β-estradiol or no induction.

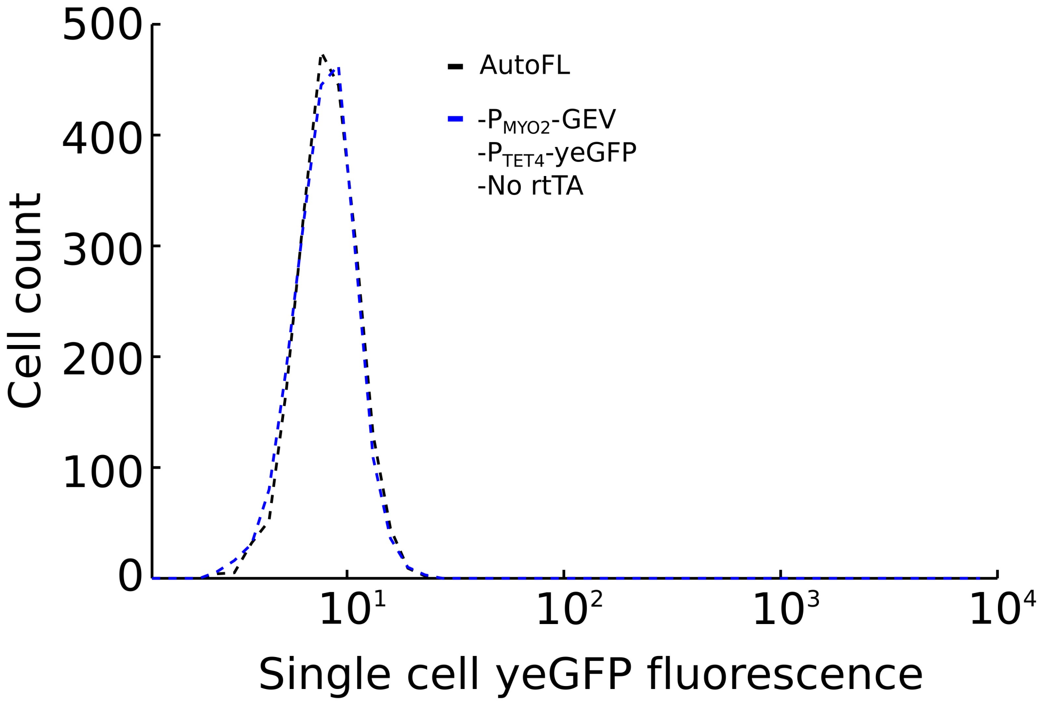

Supplemental Figure S3. yeGFP expression from the P_{TET4} promoter and GEV cassette alone is indistinguishable from autofluorescence of wildtype BY4742 yeast cells when cells were cultured in 1000nM ß-estradiol.

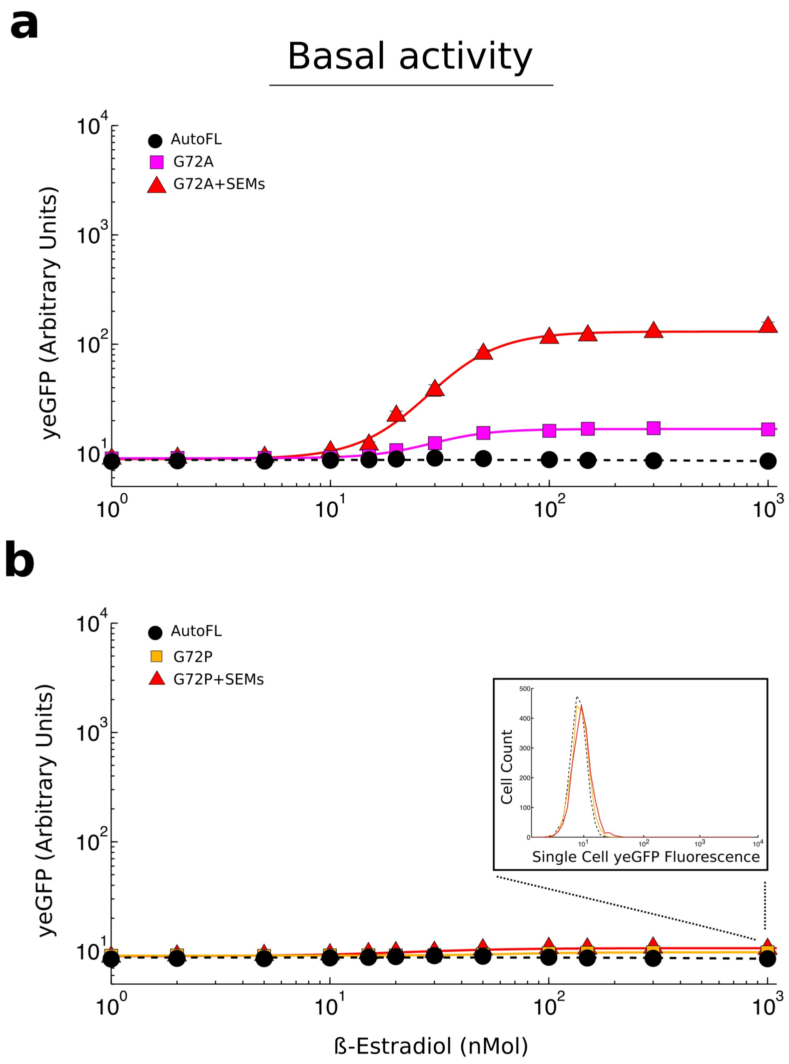

Supplemental Figure S4. **a,b,** The basal activity of rtTA variants as a function of their ß-estradiol-dependent expression level (mean +/- s.e.m of three technical replicates). In the inset of **b**, histograms of single cell fluorescence of the G72P variant and a SE-G72P variant cultured in 1000nM ß-estradiol.

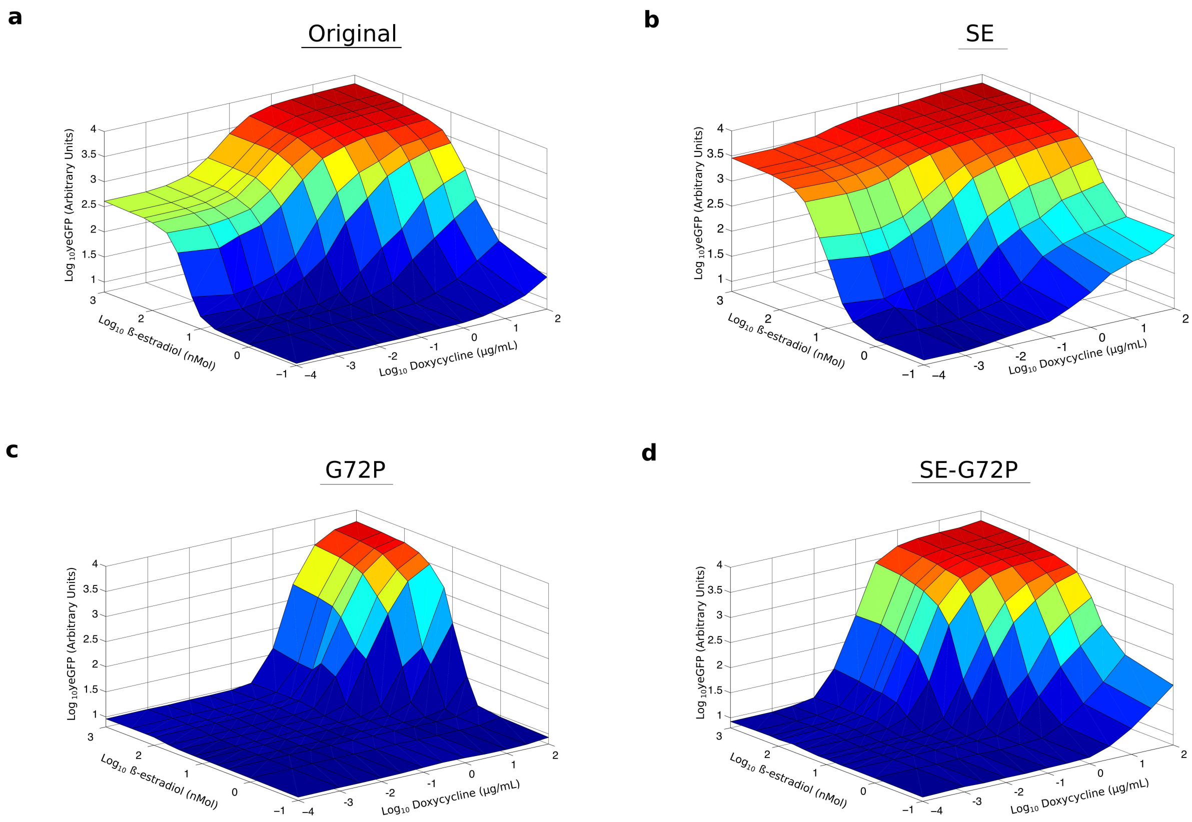

Supplemental Figure S5. Surface plots of the data presented in Figure 4 (**c-f**). **a-d**, Surface plots displaying reporter expression (Arbitrary units) as a function of β -estradiol-dependent transactivator expression and doxycycline induction.
